# Sexually divergent induction of microglial-associated neuroinflammation with hippocampal aging

**DOI:** 10.1186/s12974-017-0920-8

**Published:** 2017-07-21

**Authors:** Colleen A. Mangold, Benjamin Wronowski, Mei Du, Dustin R. Masser, Niran Hadad, Georgina V. Bixler, Robert M. Brucklacher, Matthew M. Ford, William E. Sonntag, Willard M. Freeman

**Affiliations:** 1 Department of Biochemistry and Molecular Biology, Pennsylvania State University, State College, PA USA; 2 Department of Physiology, University of Oklahoma Health Sciences Center, Oklahoma City, OK USA; 3 Reynolds Oklahoma Center on Aging & Nathan Shock Center of Excellence in the Biology of Aging, University of Oklahoma Health Sciences Center, Oklahoma City, OK USA; 4 Oklahoma Center for Neuroscience, University of Oklahoma Health Sciences Center, Oklahoma City, OK USA; 5 Genome Sciences Facility, Pennsylvania State University College of Medicine, Hershey, PA USA; 6 Division of Neuroscience, Oregon National Primate Research Center, Beaverton, Oregon USA; 7 Department of Geriatric Medicine, University of Oklahoma Health Sciences Center, Oklahoma City, USA; 8 SLY-BRC 1370, 975 NE 10th St, Oklahoma City, OK 73104 USA

**Keywords:** Sex differences, Neuroinflammation, Gene expression, Aging, Brain

## Abstract

**Background:**

The necessity of including both males and females in molecular neuroscience research is now well understood. However, there is relatively limited basic biological data on brain sex differences across the lifespan despite the differences in age-related neurological dysfunction and disease between males and females.

**Methods:**

Whole genome gene expression of young (3 months), adult (12 months), and old (24 months) male and female C57BL6 mice hippocampus was analyzed. Subsequent bioinformatic analyses and confirmations of age-related changes and sex differences in hippocampal gene and protein expression were performed.

**Results:**

Males and females demonstrate both common expression changes with aging and marked sex differences in the nature and magnitude of the aging responses. Age-related hippocampal induction of neuroinflammatory gene expression was sexually divergent and enriched for microglia-specific genes such as complement pathway components. Sexually divergent C1q protein expression was confirmed by immunoblotting and immunohistochemistry. Similar patterns of cortical sexually divergent gene expression were also evident. Additionally, inter-animal gene expression variability increased with aging in males, but not females.

**Conclusions:**

These findings demonstrate sexually divergent neuroinflammation with aging that may contribute to sex differences in age-related neurological diseases such as stroke and Alzheimer’s, specifically in the complement system. The increased expression variability in males suggests a loss of fidelity in gene expression regulation with aging. These findings reveal a central role of sex in the transcriptomic response of the hippocampus to aging that warrants further, in depth, investigations.

**Electronic supplementary material:**

The online version of this article (doi:10.1186/s12974-017-0920-8) contains supplementary material, which is available to authorized users.

## Background

Age-related changes across molecular and cellular processes likely contribute to the development of neurological disorders and functional deficits as these become more common at advanced ages. Understanding the aging process and the contribution of aging to disease and impairment of the CNS, also known as geroscience [1], offers the potential to counteract these processes and thereby prevent, slow, and possibly reverse disease and dysfunction. While we and others have examined changes in hippocampal gene expression across the lifespan in humans [2], monkeys [3], rats [4, 5], and mice [6, 7], most of these studies included only male animals, did not control for estrus cycle stage in females, or did not focus explicitly on identifying sex differences and divergences in gene expression with aging. The historical predominance of male animal models in preclinical studies [8] remains to be overcome despite efforts to include both sexes in preclinical studies [9]. For this study, the terminology recommendations stated in McCarthy et al. are followed. Sexual dimorphisms are dialectic differences between males and females (such as Y chromosome encoded gene expression is only in males). Sex differences are differences in average level of a transcript or protein that are present throughout life. Sex divergences are emergent, such as expression of a gene at young age that is the same in males and females but becomes different at old age [10].

The differential course and nature of brain aging between males and females is a much needed area of investigation as with improvements in medical care the older population (>65 years) is growing rapidly. As age increases, there is an enhanced prevalence of Alzheimer’s and other neurodegenerative diseases, as well as non-neurodegenerative cognitive impairments. Women have a higher propensity of developing Alzheimer’s disease compared to men [11, 12], a higher risk of mild cognitive impairment [13] (though not all studies are in agreement on the latter point [14]), and a lower risk of stroke but poorer outcomes after stroke [15, 16]. In support of these findings, greater age-related, non-neurodegenerative impairments of spatial learning and memory have been observed in female rats [17] and mice [18, 19] when compared to age-matched males. Furthermore, females demonstrate age-related changes in metabolic processes in the brain earlier than males [20]. Together, these data suggest differences in brain aging between the sexes that may leave females more susceptible to cognitive and neurological disorders later in life when compared to their male counterparts. It is therefore imperative that studies examine the molecular changes occurring in the brain with aging in both males and females in an effort to better determine how these changes contribute to disease and dysfunction.

Age-related gradual loss in synapse number, strength, and proper morphological phenotype occurs in both sexes. However, changes in circulating sex hormones may play a role in age-related alterations in synapse physiology. High circulating concentrations of estrogen enhance dendritic spine density, while high progesterone levels cause decreases in density [21, 22]. These data imply sex hormone changes may work through synapse regulation in causing decreases in hippocampal volume with age that have been reported to be greater in women versus men [23]. However, sex-specific differences in hippocampal volume with aging has not been consistently observed [24]. Other factors that may contribute to synaptic dysfunction with advanced age include the induction of both peripheral inflammation and local neuroinflammation [25]. We have previously reported that the major histocompatibility complex class I (MHCI) pathway is induced with advanced age in hippocampal synapses of old male rats [26] and that across the CNS MHCI (and associated genes) are more highly induced with aging in female than male mice [27]. This raises the possibility that neuroinflammation with aging may differ in nature and magnitude between the sexes. This is supported both by human gene expression data [2] and the findings that female mice display higher numbers of activated Mac-1 positive microglia in the dentate gyrus (DG) and CA1 subregions of the hippocampus when compared to age-matched males [28, 29]. The understanding of the molecular differences in the brain’s aging response is limited with no reports comparing males and females across the lifespan in in-bred, controlled animal models. To further explore sex differences in hippocampal aging, we examined gene expression in young (3 months), adult (12 months), and aged (24 months) male and female C57BL6 mice.

## Methods

### Animals

All animal experiments were executed according to protocols approved by the Penn State University Animal Care and Use Committee. Male and female C57BL/6 mice, substrain NCr (NIA colony Charles River), aged 3 (young), 12 (adult), and 24 (old) months were purchased from the National Institute on Aging colony at Charles River Laboratories (Wilmington, MA). Mice were housed in the Pennsylvania State University College of Medicine Hershey Center for Applied Research facility in ventilated HEPA filtered cages with ad libitum access to sterile food and water (Harlan 2918 diet, irradiated). In this facility, all animals are free of helicobacter and parvovirus. Following a 1-week acclimation period after arrival, male mice were euthanized by decapitation.

In female mice, estrous cycle staging was performed by daily vaginal lavage to control for cycling differences. Estrous cycle staging for all female mice was performed by daily vaginal lavage for 3–4 weeks, and animals were euthanized during diestrous. Lavages were conducted as described previously [27] and using well-established methods [30]. Briefly, sterile filtered water was expelled and aspirated approximately 4–5 times into the vaginal canal until enough cells were obtained for cytological analysis. Water from the vaginal wash was then placed onto a glass slide, allowed to dry, then stained using 0.1% crystal violet. The estrous cycle consists of three major phases: proestrus (high estrogen), estrus (low estrogen), and diestrus (low estrogen). The presence of specific cell types is indicative of each stage of the cycle. Specifically, proestrus is defined by having a predominance of round, nucleated epithelial cells, estrus by cornified squamous epithelial cells, and diestrus by leukocytes with few epithelial cells present [30].

Following euthanasia, the hippocampus (including CA1, CA2, CA3, and dentate gyrus) and cortex (somatomotor/orbital cortices, i.e., frontal cortex) were rapidly dissected. Tissues were then frozen in liquid nitrogen and stored at −80 °C until analysis. Mice used for immunohistochemical analysis were processed as previously described [31]. Animals were anesthesized with ketamine/xylazine and then transcardially perfused with 1× phosphate-buffered saline (PBS) followed by 4% paraformaldehyde buffered in 0.1 M sodium phosphate buffer (pH 7.4). Brains were then postfixed in 4% paraformaldehyde overnight at 4 °C, cryoprotected using 30% sucrose, embedded in Tissue-Tek optimal cutting temperature and then frozen in isopentane on dry ice.

### RNA isolation

RNA preparation from hippocampus and cortex was performed according to standard methods [AllPrep DNA/RNA Mini (Qiagen)] as described previously [32]. RNA quality was assessed by RNA 6000 Nano LabChip with an Agilent 2100 Expert Bioanalyzer (Agilent, Palo Alto, CA). Only samples with RNA integrity numbers greater than 7 were used in subsequent studies. RNA concentration was assessed by relative fluorescence using the RiboGreen assay (Invitrogen, Carlsbad, CA, USA).

### Microarray analysis

Transcriptomic analyses were performed on hippocampal samples derived from male and female young, adult, and old mice (*n* = 4/group, *N* = 24) using Illumina Mouse Ref8 microarrays (Illumina, San Diego, CA) according to standard methods and as previously described [5, 33]. First-strand complementary DNA (cDNA) was synthesized from 500 ng input RNA by 2-h incubation at 42 °C with T7 Oligo(dT) primer, 10× first-strand buffer, dNTPs, RNase inhibitor, and ArrayScript. Second-strand cDNA was synthesized from first-strand cDNA by 2-h incubation at 16 °C with 10× second-strand buffer, dNTPs, DNA polymerase, and RNase H, purified using the Illumina TotalPrep kit (Ambion, Foster City, CA) according to the manufacturer’s protocols and eluted in 19 μL 55 °C nuclease-free water. cRNA was synthesized from second-strand cDNA using the MEGAscript kit (Ambion) and labeled by incubation for 14 h at 37 °C with T7 10× reaction buffer, T7 Enzyme mix, and Biotin-NTP mix. Following purification with the Illumina TotalPrep RNA Amplification kit (Ambion) according to manufacturer’s instructions, cRNA yields were quantitated using a NanoDrop ND1000 spectrometer. Biotinylated cRNA (750 ng) was hybridized by incubating for 20 h at 58 °C at a rocker speed of 5. After incubation, BeadChips were washed and streptavidin-Cy3 stained, dried by centrifugation at 275×*g* for 4 min, scanned and digitized using a Bead Station Bead Array Reader.

Arrays were quality control checked, and initial data analysis using average normalization with background subtraction was performed in GenomeStudio (Illumina). The full microarray dataset has been deposited in the Gene Expression Omnibus, accession# GSE85084. Data was mean normalized and then scaled to make the median of young males 1 in GeneSpring GX (Agilent). Using detection *p* values generated by GenomeStudio, probes were filtered for only those with present or marginal calls in 100% of the samples in at least one of the six experimental groups (male, female/young, adult, or old). This ensured that transcripts not reliably detected in any group were excluded from statistical analysis and that genes potentially expressed in only one experimental animal group were retained. A two-way ANOVA design was used to identify transcripts differentially expressed with the factors of age or sex and those with interactions of the two factors. Pairwise post hoc analysis (Student–Newman–Keuls, *p* < 0.05) was performed on those genes with a significant effect (*p* < 0.05) of age, sex, or interaction effect. Rather than a global multiple testing correction, genes passing the ANOVA and post hoc statistical criteria were filtered for only those with an absolute value fold-change cutoff of |1.2| in accordance with standards for microarray analysis [34] and as previously described [26, 31, 35]. These two rounds of statistical thresholds and fold-change cutoffs were used to produce gene lists with a balance of minimizing type I and type II errors rather than a blanket false discovery rate correction which can produce a high type II error rate [36]. For variance analysis, a gene-by-gene variance (*σ* ^2^) for every gene that passed criteria as expressed was calculated for each group (age × sex). Variance between groups and data was visualized with by plotting the density of variance for each group using R package “ggplot2” version 3.3.0.

### Bioinformatic analysis and visualization

Pathway, function, regulator, and cell-specificity analyses were performed using Ingenuity Pathway Analysis—IPA (Qiagen, Redwood City, CA) software and database (March 2017 release). Cell-specific transcript lists were developed from previous reports [37, 38] (Additional file 1: Tables S1–3) and then imported into IPA for analysis of statistical over-representation. Microglial transcript signatures of classical and alternative priming genes along with the microglial sensome were derived from previous direct sequencing of microglia [39]. M0–M1–M2 phenotype gene expression markers (Additional file 1: Table S4) were derived from [40]. These gene lists were imported into IPA and compared to all gene sets from pairwise comparisons that passed statistical and fold-change cutoffs. For each pathway, process, and regulatory analyses, an overlap *p* value and an activation *z* score were computed [41]. For the cell specificity and microglial gene set analysis, only the *p* value was calculated as a *z* score is not applicable. Custom gene set lists used are provided (Additional file 1: Tables S1–4). The *p* value for enrichment of gene sets was calculated using Fisher’s exact test with Benjamini–Hochberg multiple testing correction based on overlap between genes in the list and known genes pertaining to a function, targets of a transcriptional regulator, or imported gene list. The activation *z* score is used to infer likely activation states of a function or upstream regulator based on the direction of changes in the gene list and literature-derived functional or regulation directions. A *z* score cutoff of >|2| was applied to limit lists to only those functions and regulators with considerable activation (positive *z* score) or inhibition (negative *z* score). Venn diagrams and heatmaps were generated with GeneSpring v14.5 software (Agilent).

### Quantitative PCR

cDNA was reverse transcribed with random primers from 500 ng of total RNA [ABI High Capacity cDNA Reverse Transcription Kit (Applied Biosystems Inc., Foster City, CA)] as previously described [35]. qPCR was performed with gene-specific primer probe fluorogenic exonuclease assays (Additional file 1: Table S5) (TaqMan, Life Technologies) using standard methods [5]. Relative gene expression was calculated with Expressionsuite v 1.0.3 software using the 2^−ΔΔ^Ct analysis method with β-actin as an endogenous control. Sample size for the qPCR analysis was *n* = 7–8/group for both confirmation of the microarray findings in hippocampus and extension of these targets to the cortex.

### Immunoblotting

Hippocampal tissue was solubilized in a detergent-based protein lysis buffer containing protease and phosphatase inhibitors [100 mM NaCl, 20 mM HEPES, 1 mM EDTA, 1 mM dithiothreitol, 1.0% Tween20, 1 mM Na_3_VO_4_, 1 Complete Mini EDTA-Free Protease Inhibitor Cocktail Tablet (Roche Applied Science, Indianapolis, IN, USA) for every 10 mL lysis buffer] using a bead mill (Retsch TissueLyzer II; Qiagen, Valencia, CA, USA). Homogenates were incubated at 4 °C with gentle rocking for 15 min, and insoluble protein was removed by centrifugation (10,000×*g*, 15 min, 4 °C). The soluble protein-containing supernatant was collected, and protein concentrations were determined by bicinchoninic acid quantitation (Pierce, Rockford, IL, USA).

Immnoblotting was performed according to standard methods [42, 43]. Protein samples were adjusted to a concentration of 2 μg/μL in protein lysis buffer and LDS sample buffer (Invitrogen, Carlsbad, CA, USA). Ten micrograms of each prepared protein sample was denatured at 95 °C prior to sodium dodecyl sulfate–polyacrylamide gel electrophoresis separation using Criterion Tris–HCl precast 4–20% acrylamide gradient gels (Bio-Rad, Hercules, CA, USA). An independent gel containing parallel aliquots of study samples was stained with Deep Purple total protein stain (GE Healthcare, Piscataway, NJ, USA) and quantitated by whole-lane digital densitometry (ImageQuant TL; Molecular Dynamics, Sunnyvale, CA, USA) to ensure equal protein content between samples. For immunoblotting, proteins were transferred to polyvinylidene difluoride membranes (HyBond; GE Healthcare), blocked with 3% BSA in PBS containing 1% Tween-20, and incubated with primary antibodies (Additional file 1: Table S6). Membranes were washed with PBS containing 1% Tween-20, incubated with species-appropriate secondary antibodies (Additional file 1: Table S6), and visualized with enhanced chemiluminescence substrate (GE Healthcare). Immunoreactive bands were imaged on film, digitized at a resolution of 800 d.p.i. with a transmissive scanner, and quantitated using automated digital densitometry software with rolling ball background subtraction (ImageQuant TL).

### Immunohistochemistry

Cryosections of mouse brain were sectioned 14 μm thick and processed for immunohistochemistry as previously described [44]. Briefly, cryosections were incubated in blocking solution (10% donkey serum, 5% bovine serum albumin, and 0.5% Triton X-100 in PBS) for 1 h followed by overnight incubation at 4 °C in primary antibodies (Additional file 1: Table S6) diluted in blocking buffer. Sections were then washed in PBS and incubated with appropriate fluorophore-conjugated secondary antibodies for 1.5 h. To label cell nuclei, sections were incubated with Hoechst stain at 100 ng/ml for 30 min at room temperature. Sections were then rinsed with PBS, mounted with Aqua-Poly/Mount mounting medium (Polysciences, Inc.) and covered with glass coverslips. Sections were imaged with a Nikon Eclipse Ti-U inverted research microscope. Whole brain images were taken using ×4 objective and stitched using 40% overlay.

Immunofluorescence assays of mouse brains were executed as previously described [27]. Briefly, brains were sectioned at 12 μm thick each, post-fixed in 2% paraformaldehyde, and rinsed in 1XPBS. Sections were then blocked in 10% donkey serum diluted in 0.1% Triton X-100 in PBS for 1 h at room temperature and incubated overnight at 4 °C in a humid chamber with primary antibodies to C1q and Iba1 (Additional file 1: Table S6) diluted in blocking solution. Slides were washed three times with 0.1% Triton X-100/PBS, then incubated with species-appropriate secondary antibodies (Additional file 1: Table S6) for 2 h at room temperature, protected from light. Following three washes with 0.1% Triton X-100/PBS, sections were rinsed with 1XPBS, and then coverslipped with ProLong Gold antifade mounting media (ThermoFisher). Images were acquired using an Olympus FV10i confocal microscope equipped with a ×60 water immersion objective. Background subtraction was done in ImageJ (rolling ball radius = 50).

### Statistics

All qPCR and immunoblotting data were analyzed using SigmaStat 3.5 (SyStat Software, San Jose, CA). Two-way ANOVA analyses were performed with the factors of sex and age. Post hoc testing was performed by Student–Newman–Keuls (SNK) test with *α* < 0.05. For qPCR and immunoblotting analysis, a Benjamini–Hochberg multiple testing correction was applied to the *F* test result to correct for the number transcripts analyzed, and for immunoblotting, the correction was applied to the number of proteins examined. Multiple testing correction was applied to hippocampal and cortical analyses separately. Gene expression variance was analyzed by two-way ANOVA with SNK post hoc of the inter-animal variances in each group for each gene.

## Results

To identify age and sex differences in hippocampal gene expression, male and female mouse samples from ages 3 months (young), 12 months (adult), and 24 months (old) were compared by microarray analysis. Estrous cycle stage of the female mice was monitored daily for 3–4 weeks and all female mice were sacrificed during diestrus. Old female mice were confirmed to be in reproductive senescence (permanent diestrus).

### Age-related gene expression changes in males and females

**Fig. 1:**
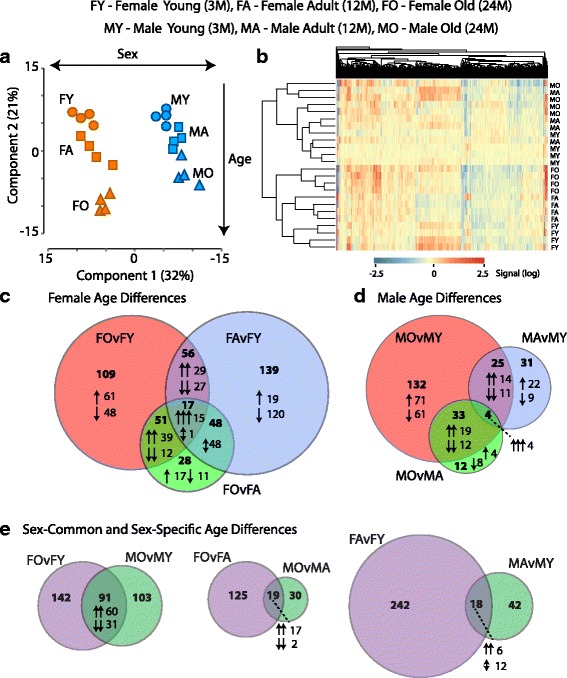
Hippocampal gene expression changes with aging. **a** Principle component analysis (PCA) of individual samples using all expressed genes. Groups separated by sex along the 1st component and by age in the 2nd component. **b** Heatmap presentation of all age-related gene expression differences. Samples were clustered (Euclidean distance) by sex and age. **c** Comparison of pairwise age-related changes in females. Total numbers of genes and direction of change (induction/reduction). For intersections patterns in respective groups are also noted. **d** Venn diagram of pairwise aging changes in males. **e** Comparison of age-related differences between males and females

Of the 25,697 probes on the microarray, 9540 passed filtering as expressed in at least one of the experimental groups. To compare the overall gene expression profiles between groups, a principal component analysis (PCA) was performed on the groups using the full set of 9540 expressed genes (Fig. 1a). Individual samples segregated (female young—FY, female adult—FA, female old—FO, male young—MY, male adult—MA, male old—MO) by sex in first component by sex and by age in the second component. Female mice showed a larger shift in global gene expression profile with aging when compared to age-matched males. To further assess age and sex-specific differences in gene expression with aging, a two-way ANOVA (factors of sex and age) was performed with pairwise Student–Newman–Keuls post hoc testing on all expressed genes. Genes passing ANOVA and SNK post hoc were further filtered for only those with |>1.2| fold change in the specific significant pairwise comparison. Five hundred sixty-four genes in total were significantly altered in expression with aging in females and/or males as visualized in the heatmap presented in Fig. 1b and are given in Additional file 1: Table S7. Clustering of the individual samples showed a separation by sex and then age similar to that observed with the PCA. Comparing the pairwise age-related differences in females, commonly regulated genes in multiple pairwise comparisons (Fig. 1c) were generally consistent in the direction of change across comparisons. The exception being the 48 transcripts in the intersection of FA versus FY and FO versus FA, as these are not differentially expressed in the FO versus FY comparison and demonstrated a “V” or “inverted V” expression pattern across the lifespan. In males, differences with age were consistent across pairwise comparisons (Fig. 1d). Age-related changes in gene expression showed some commonalities between sexes (Fig. 1e) and were almost always coordinately regulated with age in males and females. However, the majority of age-related changes were sex specific. Comparisons across all six pairwise comparisons are presented in Additional file 1: Figure S1.

**Fig. 2:**
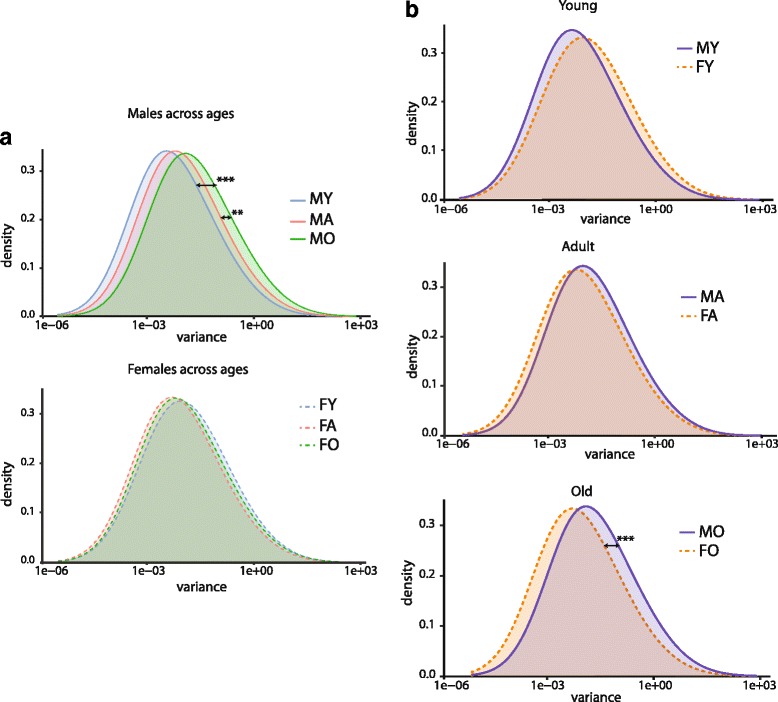
Gene expression variance with aging. **a** The distribution of inter-animal variance across ages was compared for females and males. Increasing variance with age was evident in males but not females. **b** Pairwise comparisons of variance between males and females at each age. Two-way ANOVA, SNK **p < 0.01, ***p < 0.001

Previous studies have also reported increased variability of gene expression with aging at the cell-to-cell [45] and inter-animal (animal-to-animal) [46, 47] levels, suggestive of a loss of tight transcript control with aging. Inter-animal variance (*σ* ^2^) was computed for each expressed gene within each group. Examining the distribution of variance in males and females across the lifespan, an increase in gene expression variance was evident with aging (two-way effect of age and interaction of age and sex) (Fig. 2a). Post hoc testing (SNK) reveals an increase in expression variance in old males as compared to young and adult males. No differences with age in variance were observed in females. Comparing differences in variance between sexes at each age, male gene expression was more variant than females in old age but not different at young and adult ages (Fig. 2b).

**Fig. 3:**
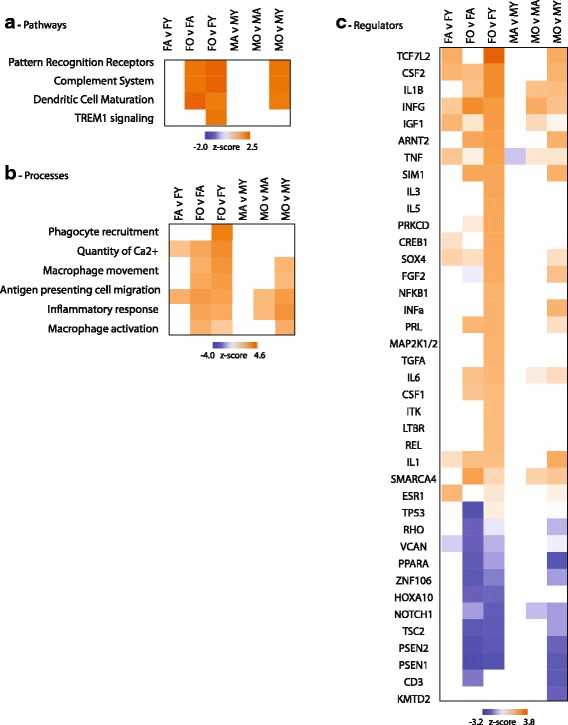
Pathway, function, and regulatory analysis of age-related transcriptomic changes. Age-related gene expression changes were analyzed with Ingenuity Knowledge Base for differentially regulated pathways (**a**), functions (**b**), and regulators (**c**). A relevant selection of over-represented categories (Fisher’s exact test *p* < 0.05) is given in heatmap form with coloring according to the computed *z* score. *Z* scores are based on prior knowledge of known regulatory functions and direction of changes in the current dataset. *Z* scores >2 indicate significant activation with aging and <−2 indicate significant inhibition with aging. Abbreviations are detailed in Additional file 1: Table S8

To place age-related changes in gene expression into a biological context, each set of pairwise aging differences was analyzed for over-representation of pathways (Fig. 3a), processes (Fig. 3b), and regulators (Fig. 3c). Activation of inflammatory pathways was evident in both females and males with aging. Importantly, in females, these changes are evident in both young versus old (FY vs FO) and adult versus old (FA vs FO) comparisons indicating a more pronounced activation later in the lifespan (Fig. 3a, b). Common upstream regulators were also evident with aging (Fig. 3c), including a number of pro-inflammatory factors. Full lists of pathways, regulators, and processes are in Additional file 1: Table S8.

**Fig. 4:**
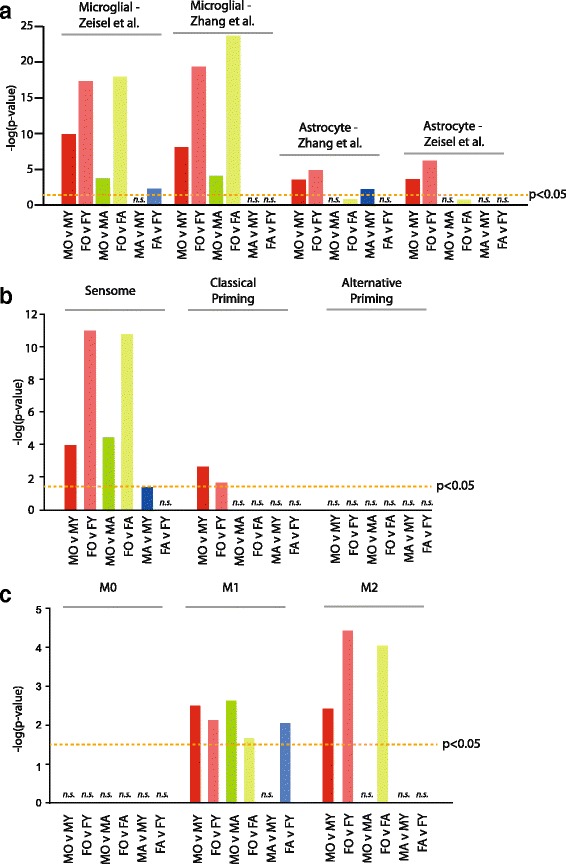
Enrichment of age-related changes in cell-specific transcripts. **a** Cell-specific transcripts from previous reports (Zeisel et al. [30] and Zhang et al. [31]) were compared to each pairwise set of age-related changes. Fisher’s exact test *p* values are plotted for cell types with significant over-representations. **b** Using gene sets derived from Hickman et al. [32] for the sensome, classical priming, and alternative microglial priming, a significant over-representation of sensome genes, in particular, is evident. **c** Previously published gene sets indicative of M0, M1, and M2 microglial states [33] were also examined for over-representation of age-related genes

Recent studies have begun to define sets of genes that are solely expressed/highly enriched (e.g., >10 fold) in specific cell types using RNA sequencing of individual cells with post hoc definition of the individual cell’s identity [37] or purification of individual cell types followed by RNA sequencing [38] (Additional file 1: Table S1–3). Using cell-specific gene sets for neurons, astrocytes, microglial, endothelial, mural, oligenodrocyte, and other cells as references, it is possible to determine if there are more age-related changes arising from specific cell types than would be expected by random chance. In the current dataset, a high level of enrichment in microglia-specific and to a lesser extent astrocyte-specific genes was evident for age-related changes in gene expression (Fig. 4a) but not of other cells types examined.

Similarly, recent work has sought to place microglial genes in further subsets such as the sensome, classical priming, and alternative priming. The microglial “sensome” is defined as a distinct set of messenger RNA (mRNA) transcripts that encode for proteins involved in microglial sensing of endogenous ligands and pathogens [39]. Sensome genes were highly over-represented in age-related changes with limited or no enrichment for classical and alternative priming genes, potentially indicating an altered surveilling state with aging but not consistent with a prototypical microglial priming response (Fig. 4b). This is further demonstrated by an enrichment of both M1 and M2 activation state markers (from [40]) in males and females with aging (Fig. 4c).

### Sex differences in hippocampal gene expression across the lifespan

**Fig. 5:**
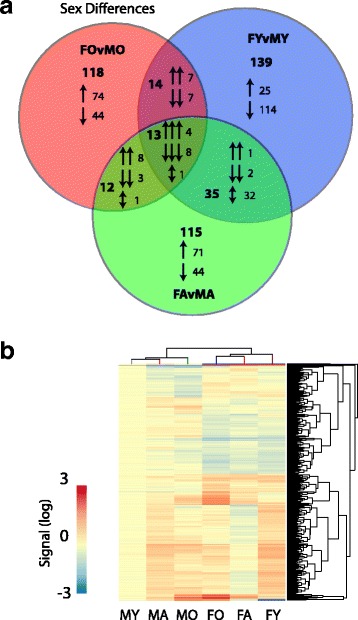
Hippocampal gene expression sex differences across the lifespan. **a** Sex-differences at each age are compared with the number of genes and direction of change (induction/reduction) noted. **b** Heatmap presentation of all sex differences in gene expression

**Fig. 6:**
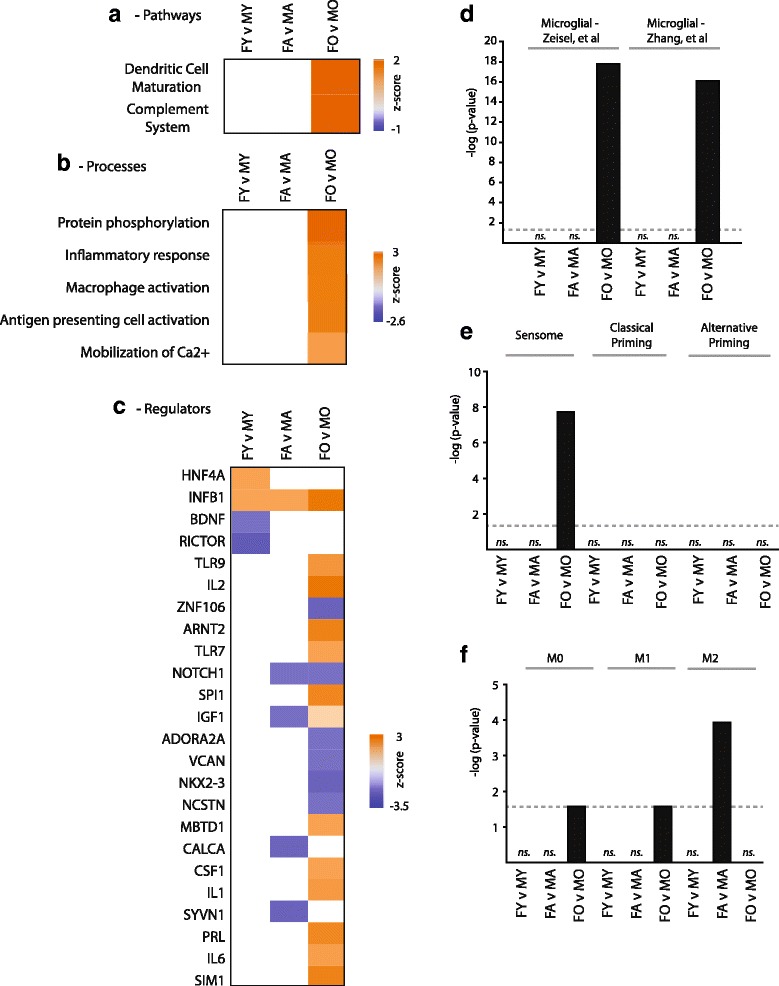
Pathway, function, and regulatory analysis of sex differences in gene expression. A selection of statistically over-represented pathways (**a**), functions (**b**), and regulators (**c**) are presented with *z* scores is given in heatmap form with coloring according to the computed *z* score. *Z* scores are based on prior knowledge of known regulatory functions and direction of changes in the current dataset. *Z* scores >2 indicate significant activation in females as compared to males and <−2 indicate significant inhibition in females compared to males. **d** Cell-specific transcripts from previous reports [30, 31]) were compared to each pairwise set of sex differences. Fisher’s exact test *p* values are plotted for cell types with significant over-representations. Gen sets derived for the sensome, classical priming, and alternative microglial priming [32] (**e**),and gene sets indicative of M0, M1, and M2 microglial states (**f**) [33] were also examined for over-representation of age-related genes. Abbreviations are detailed in Additional File: Table S10

The above analyses have focused on age-related changes in gene expression but sex-differences within each of the ages were also examined. For genes which were statistically significant for sex as a factor or showed an interaction of sex and age, pairwise sex comparisons within each age were also performed and filtered for fold differences |>1.2|. Most sex differences were found to be age specific (Fig. 5a) with those changes common across ages being coordinately regulated. Sex differences are presented in heatmap form in Fig. 5b and full gene lists are in Additional file 1: Table S9. Analyzing sex differences in expression for over-representation of pathways, processes, and regulatory factors (Fig. 6a–c) reveals primary effects at old age with females demonstrating positive *z* scores, indicating activation, for inflammatory processes as compared to old males (Additional file 1: Table S10). When examining overrepresentation of genes expressed by a single cell type, sex differences in old age were highly enriched for microglia-specifc genes (Fig. 6d) and sensome genes in particular (Fig. 6f). Additionally, enrichment of M0 and M1 marker genes was evident in old animal sex differences with M2 markers enriched in sex differences at the adult age (Fig. 6f).

### Confirmation of differential gene and protein expression

**Fig. 7:**
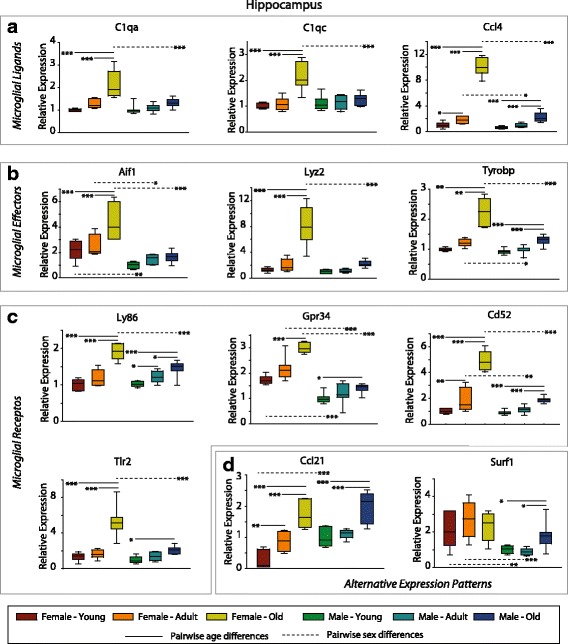
qPCR confirmation of differential sex- and age-related hippocampal gene expression. Selected microglial ligands (**a**), effectors (**b**), and receptors (**c**) targets identified in the microarray study were confirmed by gene-specific qPCR. Data is scaled to a mean value of 1 for young males. Boxes boundaries are the 25th and 75th percentiles, with median denoted by the bar and error bars at the 10th and 90th percentiles. Two-way ANOVA (age × sex), ***p < 0.001, **p < 0.01, *p < 0.05 Student–Newman–Kuels pairwise post hoc, *n* = 7–8/group. ANOVA values are presented in the text. *Solid comparison lines* denote age-related changes with a sex and *dashed comparison lines* are sex-related differences within an age. **d** A selection of genes with alternate expression parameters were also confirmed

With the clear enrichment of both sex- and age-dependent changes in hippocampal gene expression of microglial and inflammatory genes, a selection of microglial ligands (C1qa, C1qc, and Ccl4; Fig. 7a), effectors (Aif1, Lyz2, Tyrobp; Fig. 7b), and receptors (Ly86, Gpr34, Cd52, Tlr2; Fig. 7c) that were differentially expressed from the microarray analysis were confirmed by qPCR. These results confirm the microarray findings by an orthogonal method in a larger set of samples and demonstrate the sexually divergent nature of age-related changes. For all transcripts examined, there was an age-related induction in females, while in males, smaller magnitude increases in expression were evident (Ccl4, Tyrobp, Ly86, Gpr34, Cd52, Tlr2). The increases in expression with age in females was greater than in males, resulting in sex differences at old age but not in young animals (C1qa, C1qc, Ccl4, Lyz2, Tyrobp, Ly86, Cd52, Tlr2). Genes with alternate expression patterns seen when comparing sexes and with aging were also confirmed including Ccl21 where a sex difference at young age dissipates with age-related induction in both males and females, and Surf1 with an age-related increase in only males (Fig. 7d). Expression of X and Y chromosome genes, Xist, and Jarid1d, respectively, were analyzed as positive controls for sex differences/dimorphisms, as well as Dlgh4 as a negative control, a gene that demonstrated no age or sex-dependent changes in expression (Additional file 1: Figure S2).

**Fig. 8:**
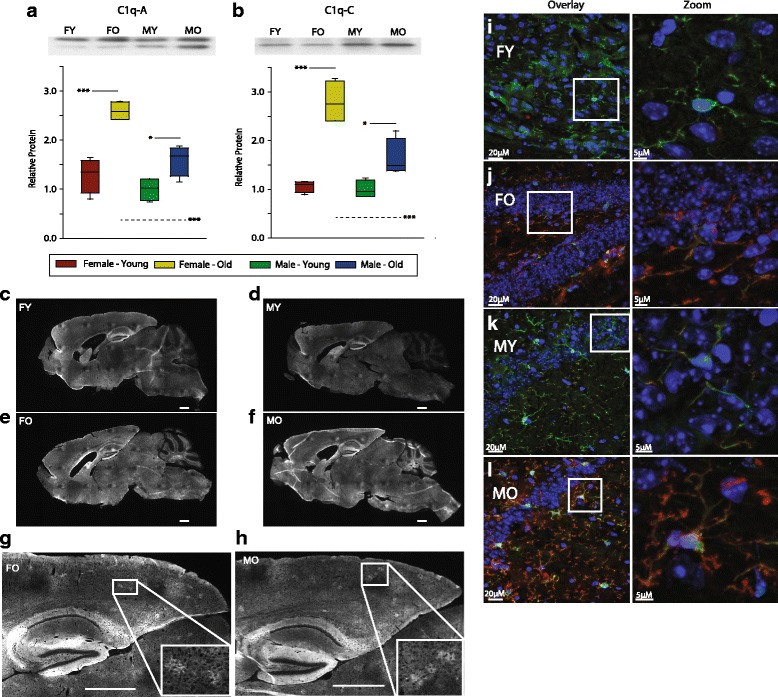
Sexually divergent, age-related hippocampal C1q protein expression. Protein expression of compliment 1q isoforms C1qA (**a**) and C1qC (**b**) were induced with age and to a greater extent in females than males. Data is scaled to a mean value of 1 for young males. Boxes boundaries are the 25th and 75th percentiles, with median denoted by the bar and error bars at the 10th and 90th percentiles. Two-way ANOVA (age × sex), ***p < 0.001, *p < 0.05 Student–Newman–Kuels post hoc, *n* = 6/group. *Solid comparison lines* denote age-related changes with a sex, and *dashed comparison lines* are sex-related differences within an age. In sagittal brain sections qualitatively increased immunoreactivity for C1q was evident with aging across the brain in both female and male mice (**c**–**f**). Detail regions of 24-month-old female (**g**) and male (**h**) mice show patches of C1qa-positive signals throughout the brain neuropil. *Boxed area* shows further magnified image to show the details of C1qa-positive patches. *Scale bars* 1 mm. C1q immunoreactivity was co-localized with Iba1 immunoreactivity in young females (**i**), old females (**j**), young males (**k**), and old males (**l**) demonstrating microglial expression. *Scale bars* 20 μm wide view, 5 μm zoomed view

To further confirm these findings at the protein level, expression of C1qa and C1qc were examined in the hippocampus in the same set of male and female, young, and old animals by immunoblotting. Concurrent with gene expression, age-related increases in C1q protein expression were evident in both females and males (Fig. 8a, b). Increased protein expression with aging was greater in females than males resulting in a sex difference in old animals. C1q expression was qualitatively greater throughout the brain as visualized by immunoreactive protein in both females and males with aging (Fig. 8c–f). Recently, a proteomic analysis of isolated microglia from young (3–5 M) and old (20–24 M) mice was reported [48]. Comparing the proteins found to be differentially expressed with aging and the transcripts observed here, common induction of Dync1l2, Gltp, Tcirg1, Mobp, Ctsz, Iba1, Ly86, Cyba, and H2-D1 were observed in both studies, with only Fgd2 demonstrating opposite regulation, providing further support that the transcript changes observed here are reflected at the protein level.

Localization of protein expression was also examined, and with aging, patches of C1q immunoreactivity were evident in males and females (Fig. 8g, h), as previously reported [49]. Continuing in this examination, C1q, with a different antibody, was co-localized with Iba1 in young and old males and females (Fig. 8i–l). This further demonstrated increased qualitative levels of C1q immunoreactivity with aging and the co-localization of this signal with the microglial marker Iba1.

**Fig. 9:**
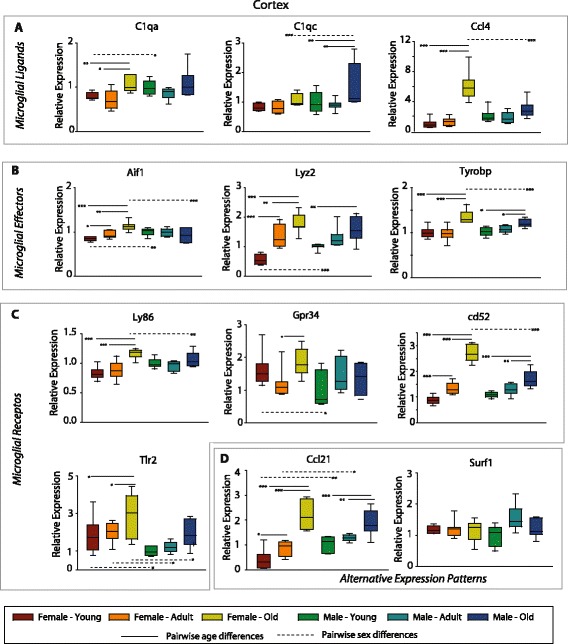
Examine of sexually divergent gene expression in the cortex. To examine whether sexually divergent age-related changes in gene expression are evident across brain regions, selected microglial ligands (**a**), effectors (**b**), and receptors (**c**) targets from the hippocampus were examined in the cortex. Data is scaled to a mean value of 1 for young males. Boxes boundaries are the 25th and 75th percentiles, with median denoted by the bar and error bars at the 10th and 90th percentiles. Two-way ANOVA (age × sex), ***p < 0.001, **p < 0.01, *p < 0.05 Student–Newman–Kuels pairwise post hoc, *n* = 7–8/group. *Solid comparison lines* denote age-related changes within a sex, and *dashed comparison lines* are sex-related differences within an age. **d** A selection of genes with alternate expression parameters were also confirmed

Lastly, to examine whether these sexually divergent aging responses were evident in other brain regions, the same set of microglial ligands, effectors, and receptors was examined in cortex samples from the same animal set. Significant pairwise differences are presented in Fig. 9a–d. Cortical patterns were similar to those in the hippocampus, with in many instances, a higher level of induction evident in females vs. males. However, this was not true for all of the genes examined.

## Discussion

### Summary of results

Consistent with previous data from human samples [2], the studies presented here demonstrate an age-related induction of inflammation-related gene expression in both the hippocampus and cortex in the brains of aged male and female mice. Importantly, changes in inflammatory genes were amplified in females, resulting in sex divergences at old age—i.e., enhanced expression of inflammation-related transcripts when compared to age-matched males. Genes regulated with aging were highly enriched for microglia-specific transcripts, and particularly members of the complement pathway and the microglial “sensome” [39]. Together, these data suggest that while there are sex-common changes with aging in the hippocampus, there is a significant difference in the nature and magnitude of neuroinflammatory changes between sexes. These effects of sex are also manifested in an increase in inter-animal gene expression variability with aging in males that is not observed in females.

### Microglial activation with aging and sex differences

Microglia serve as the first line of defense in the CNS by protecting the local environment against invading pathogens, helping recover from injury, and also playing significant roles in synapse pruning and neurodevelopment [50]. At homeostasis, microglia continuously monitor the surrounding environment and as such, maintain a ramified morphology with numerous long processes that project out from the cell body. Upon activation by the presence of an external pathogen, inflammation, or injury, microglial morphology changes, and movement to sites of injury or stress can occur along with a release soluble immune mediators [51, 52].

Traditionally activated microglia have been split into two distinct groups: M1 (classical) and M2 (activation/deactivation) [52, 53]. Classical, M1 activation is triggered by the presence of foreign antigen or pro-inflammatory cytokines, whereby microglia become more cytotoxic and release additional pro-inflammatory cytokines and free radicals [54, 55]. Alternative activation (M2) of microglia yields a more anti-inflammatory, neuroprotective phenotype that is important in the transition between a classical inflammatory response, to a decrease in inflammation [52, 54]. These microglia secrete anti-inflammatory cytokines and neurotrophic factors and help repair local damage [52]. Despite the anti-inflammatory nature of M2 microglia, the irregular abundance of both M1 and M2 type microglia may underlie chronic neuroinflammation and parainflammation, with aging [52, 56]. In support of this, using an Alzheimer’s disease mouse model, a distinct shift in activated microglia phenotypes occurs between the beginning of Aβ pathology (alternative phenotype) and advanced stages (classical phenotype), the latter of which may cause disease-associated neuron loss [57]. As such, aberrant induction or changes in the ratios of M1 and M2 activated microglia with increasing age may be maladaptive. However, the idea of M1 and M2 activation states may be too simplistic [58]. These maladaptive responses may be due to miscommunication between damaged neurons and microglia causing persistent parainflammation [59, 60] and failure of appropriate responses to different stimuli [60] that can switch from being neuroprotective to neurotoxic with aging [61]. This altered response pattern with aging is observed in response to pathogens [62], and injury [63]. Together, these data suggest that with advanced age, microglia are undergoing activation and alteration, potentially with a shift from neuroprotection to neurotoxicity. More broadly, these findings add aging to the variety of stimuli that demonstrate a sexually divergent or dimorphic neuroinflammatory response [64, 65].

Previous focused examinations have found sex differences at early ages in selected microglial genes at ages equivalent to the young and adult ages examined here [66]. We have demonstrated distinct differences in the induction of MHCI pathway genes in the brains of aged male and female 24-month-old mice, where aged females exhibit significantly higher expression [27] when compared to males, a finding with support in human datasets [2]. The findings here expand the analysis to the broader transcriptome and identify an enrichment of microglial-specific genes in age changes and sex differences. Many of the neuroinflammatory genes changed in expression with aging were common between the sexes with females demonstrating greater magnitude changes. The sexually divergent induction of Tyrobp is of special interest give the recent identification of Tyrobp, also known as TREM2, as a causal regulator in microglia associated changes in AD [67] through the exact mechanistic role of Tyrobp in AD etiology is still being determined [68]. Confirmation of selected microglial ligands, effectors, and receptors validates this pattern of gene expression. Reproducibility of expression signatures for microglial aging with previously reported data suggests a robustness to this phenomenon [69] though this study is the first to our knowledge to examine sex differences with aging in detail. Selected transcripts were also found to be sexually divergent in the cortex with some differences as compared to the hippocampus, indicative of the microglial heterogeneity observed between brain regions [70].

Our findings demonstrate that neuroinflammation with aging may represent a pattern presents a phenotype more complex than the previous hypotheses of microglial as existing in activated or resting. These states may be too simplistic, with microglial having surveilling, classically activated/M1, and alternatively activated/M2 states or an even more complex combination of activational states and not all microglia in a brain region being in the same state [39, 71, 72]. Future studies examining isolated microglial cells with new high-throughput single cell technologies [73] would greatly extend these findings to determine if these patterns are shared across individual microglial cells, or if the activation is heterogenous. Additionally, interventional studies to determine if these changes are positively adaptive or maladaptive are needed, as well as examinations of the regulation of age-related changes by sex hormones or non-sex hormone mediated mechanisms [74].

A potential concern with these findings is the effects of a change in microglial that microglia cell numbers with age. Changes in the number of hippocampal microglial with age remain an unresolved controversy. Studies have reported no changes in microglial number in mice [29] and rats [31], decreased microglial number [75], and increased microglial number in females but not males with aging [28]. Microglial quantitation was not a goal of this study but clearly is an important question to be resolved in the field and if there are changes in microglial population numbers they could play a role in the findings presented here. Arguing against this interpretation are the findings of similar patterns of gene induction in isolated microglial from aged mice [39], an experimental design that would normalize out differences in cell number. Ultimately, detailed analysis of microglial number and activation state with aging in both females and males are needed [76] and application of single cell analysis techniques will allow further refinement of these findings.

### Complement pathway and neuroinflammation

Previous reports have detailed alterations in neuroinflammation in the aged brain (as reviewed in [25]) as well as the participation of cellular senescence in the pathogenesis of brain aging [77]. A notable finding presented here is the significant induction in expression of complement pathway components in both males and females but to a much greater extent in females, in the hippocampus with advanced age. These findings are supported by data in the aged human hippocampus [78] and in studies in male mice [49]; however, to date, no between sex comparisons has been conducted. Previous work has generally examined sexually divergent differences in gene expression in the brain with aging comparing the number of gene expression changes in both males and females and separating gene expression profiles based on up or downregulation [2]. The study presented here used a more holistic approach and examined over-representation of classes of genes as well as both inductions and reductions in gene expression that may act synergistically.

Recent evidence has shown the importance of complement pathway components including C1q and C3 in activity-dependent synaptic refinement during development [79–82]. Complement factors expressed in the brain effectively label cells that need to be eliminated by local complement receptor-expressing microglia, including weak synaptic inputs [79, 80]. In response to a pathogen, West Nile Virus, C1qa induction is a driver of synapse loss with greater C1qa induction associated with poorer cognitive performance [83]. Given the role of complement pathway components in the homeostatic regulation of synapse formation and health, aberrant expression of complement proteins may play a significant role in synapse loss with aging and neurodegenerative disease [80, 82]. Previous studies have demonstrated an induction in the expression of complement factors with advanced age in both the aged mouse neocortex and cerebellum [6] and the aged rat striatum [84] as well as in neurodegenerative disease (as reviewed in [85]). Recently, complement pathway factors have been shown to play strong roles in synapse loss with normal aging [86] and the pathogenesis of neurodegenerative disease [87]. This suggests that aberrant neuron–microglial communication via the complement pathway leads to inappropriate synapse loss which may lead to cell death and the manifestation of neurodegenerative disease [80]. In further support of this, findings from a mouse model of glaucoma demonstrated elevated C1q expression is evident in adult retinal synapses prior to neuron cell death, suggesting aberrant expression of complement components leads to synapse loss and disease progression [80, 82].

Age-related complement C1q induction with aging has previously been described in male rodents and in human brain [49]. Little data exists detailing sex divergences in inflammatory gene expression in the brain. In the human brain, a higher basal level of complement cascade genes and interleukin 1 receptor-like 1 (IL1RL1) was evident in women versus men [88]. However, to date, no studies have directly described a sexually divergent neuroinflammatory response with aging. The data presented here demonstrates a heightened neuroinflammatory profile in aged female mice in comparison to males. This is true at mRNA and protein levels and can be seen across the brain with patches of C1q immunoreactivity developing with aging, that have previously been demonstrated to overlap with microgial markers [49].Elevated levels of complement pathway components and other immune factors may cause aberrant synapse elimination mediated by microglia potentially underlying the sexually divergent hippocampal volume loss seen in humans with aging [23]. Together, these data suggest sex may be a risk factor for the development of immune related diseases and CNS neuroinflammation [23, 89–91], specifically post-menopause when estrogen levels drop [92]. These sex dependent biases seen in gene expression may possibly be driven by differences in circulating sex hormones, sex-specific developmental program, or direct actions of sex chromosomes [93]. As such, including females in preclinical geroscience research studies is imperative in order to develop a full understanding of the “sexome” [94] with brain aging.

#### Other pathways and expression entropy with aging

In addition to the microglial and neuroinflammatory findings, significant decreases in the activation of both Notch1 and Presenilin 1 and 2 (PSEN1, PSEN2) regulated genes with aging were evident in both males and females. Importantly, both pathways are associated with neurogenesis. Specifically, Notch1 expression is necessary for neural stem cell maintenance [95] while PSEN1 expression regulates neuroprogentor cell differentiation [96]. Notch1 expression has previously been reported to be downregulated in the subventricular zone (SVZ) with aging [97]. Additionally, defects in PSEN1 expression are associated with the manifestation of Alzheimer’s disease in old age [98]. Decreased expression of these pathways may contribute to the known impairment of neurogenesis with aging in the hippocampus [99]. It is also important to note that microglia play important roles in neurogenesis [100, 101]. As such, the altered microglia-derived gene expression and the inhibition of pro-neurogenesis pathways evident with aging in the present study could be interrelated [102].

Another finding from the present study was decreased expression of tuberous sclerosis complex 2 (TSC2) regulated genes in both males and females with advanced age, and also in aged females when compared to age-matched males. TSC2 forms a complex with TSC1, and together, the complex functions to inhibit the mammalian target of rapamycin (mTOR) [103]. mTOR serves as a master regulator of many cellular processes including protein synthesis, proliferation, and cell survival. In the brain, mTOR has a multitude of different functions such as neuronal development, growth of dendrites and axons, neuronal migration, synaptic plasticity, neurotransmission, and DNA repair (see review [104]). Importantly, aberrant expression of TSC1 or TSC2 causes significant neurological disease, and overactivation of mTOR has been linked to the development of neurodegenerative disorders [103]. mTOR is a strong negative regulator of autophagy. As such, dysregulated mTOR activity following decreased TSC2 expression may lead to increased protein aggregation and decreased autophagy. Pharmacological inhibition of mTOR via rapamycin treatment has shown increases in life span [105] and neuroprotection [106], suggesting dysregulated mTOR signaling with age may contribute to brain aging. However, evidence exists documenting the requirement of mTOR in the development of proper dendritic arbor morphology [107] and in the stress-induced induction of post-synaptic density 95 (PSD-95) protein expression [108], hypothesized to underlie long-term potentiation (LTP) and long-term depression (LTD). These data highlight the need to study alterations in mTOR activity and responsiveness in both young and aged population to better understand aberrant activity with age.

The finding of increased inter-animal gene expression variance with aging in males but not females provides a different view on hippocampal gene expression with aging. Given that the mice used in this study (C57BL/6) are inbred and spent their entire lives under the same controlled conditions, males demonstrated an increased animal-to-animal variance with aging that was not evident in females. Previously increased cell-to-cell variability of gene expression in cardiomyocytes [45] with aging has been reported, as well as animal-to-animal increases in gene expression variance in a variety of tissues in males [46, 47, 109]. We observe that males steadily increase in variance across the lifespan while females do not, ultimately resulting in a higher level of inter-animal variance in old age in males as compared to females. The only report we are aware of examining males and females also found that inter-animal variance increased only in males [109]. The functional implications of this difference are not clear, but this may be a result of underlying epigenetic changes [110]. Confirmation studies across multiple tissues and with higher sample numbers are needed to explore this intrinsic variability with aging in males. Lastly, for both the sex divergences in gene expression and the increased variance in gene expression only observed in males, future studies will need to dissect the causes of these differences at the level of development, direct action of gonadal hormones, or sex chromosomes [93] and whether these age-related alterations are associated with cognitive impairment [111].

## Conclusions

The results presented here demonstrate that aged females experience a distinct difference in brain aging when compared to age-matched males, suggesting females undergo a higher level of microglial activation with age. These data have significant implications on the molecular mechanisms underlying brain aging, and the development of neurodegenerative disease in males and females, highlighting the importance of studying both sexes in geroscience research. This study did not seek to mechanistically explain sexually divergent responses with aging. Future studies, preferably from isolated cell types or single cells, are needed to address the origin of these sex-specific responses in gene expression. Additionally, examinations of the functional implications of sexually divergent aging responses are needed. Nonetheless, these data provide a compelling rationale for the inclusion of both female and male rodents in basic aging research and offer important new avenues for future investigation.

## Additional files

Additional file 1: Figure S1. Comparison of all pairwise gene expression sets. **Figure S2.** qPCR controls. **Table S1.** Cell-specific gene lists from Zhang et al. **Table S2.** Cell-specific gene lists from Zeisel et al. **Table S3.** Microglial gene lists from Hickman et al. **Table S4.** Gene Expression Assays. **Table S5.** Primary and secondary antibodies. **Table S6.** Transcripts differentially expressed with age. **Table S7.** Pathway, regulator and function changes with aging. **Table S8.** Transcripts differentially expressed between sexes. **Table S9.** Pathway, regulator and function differences between sexes. **Table S10.** Sex difference pathways, processes, and regulators. (ZIP 1133 kb)

## Abbreviations

BHMTC: Benjamini–Hochberg multiple testing correction
SNK: Student–Newman–Keuls
