# Supplementary material for: Sexually divergent induction of microglial-associated neuroinflammation with hippocampal aging
Source: J Neuroinflammation. 2017 Jul 21;14:141. doi: 10.1186/s12974-017-0920-8 (PMC5521082; doi:10.1186/s12974-017-0920-8)
Supplement: Supplementary file 1 — Comparison of all pairwise gene expression sets. Figure S2. qPCR controls. Table S1. Cell-specific gene lists from Zhang et al. Table S2. Cell-specific gene lists from Zeisel et al. Table S3. Microglial gene lists from Hickman et al. Table S4. Gene Expression Assays. Table S5. Primary and secondary antibodies. Table S6. Transcripts differentially expressed with age. Table S7. Pathway, regulator and function changes with aging. Table S8. Transcripts differentially expressed between sexes. Table S9. Pathway, regulator and function differences between sexes. Table S10. Sex difference pathways, processes, and regulators. (ZIP 1133 kb) [file 12974_2017_920_MOESM1_ESM.zip › Additional files/Supplemental Table 5 - Primers.docx]

Table S1 – Primer/probe sets used in gene expression analysis.

| **Gene symbol** | **Gene ID number** | **Gene name** | **Gene expression assay catalog #** | **Alias** |
| --- | --- | --- | --- | --- |
| Tyrobp | 22177 | Tyrosine Kinase Binding Protein | Mm00449152_m1 | Ly83; DAP12; KARAP |
| Ccl4 | 20303 | Chemokine (C-C Motif) Ligand 4 | Mm00443111_m1 | AT744.1, Act-2, MIP-1B, Mip1b, Scya4 |
| Cd52 | 23833 | CAMPATH-1 antigen | Mm00489055_m1 | AI463198, B7, B7-Ag, CAMPATH-1, CLS1, MB7 |
| Xist | 213742 | X Inactive Specific Transcript | Mm01232884_m1 | A430022B11, AI314753 |
| Gapdh | 14433 | Glyceraldehyde-3-Phosphate Dehydrogenase | Mm99999915_g1 | Gapd |
| C1qa | 12259 | Complement Component 1, Subcomponent, A Chain | Mm00432142_m1 | C1q; AI255395 |
| C1qc | 12262 | Complement Component 1, Subcomponent, C Chain | Mm00776126_m1 | C1qg; Ciqc; AI385742 |
| Ly86 | 17084 | Lymphocyte Antigen 86 | Mm00440240_m1 | MD1; MD-1; ly86_tv2 |
| Lyz2 | 17105 | Lysozyme 2 | Mm01612741_m1 | Lys; Lzm; Lzp; Lysm; Lyzs; Lyzf2; Lzm-s1; AI326280 |
| Aif1 | 11629 | Allograft Inflammatory Factor 1 | Mm00479862_g1 | 0610010F23Rik, Surf-1 |
| Surf1 | 20930 | Surfeit 1 | Mm00489041_g1 | Surf-1; 0610010F23Rik |
| Kdm5d | 20592 | Lysine (K)-Specific Demethylase | Mm00803406_m1 | HY, Jarid1d, Smcy |
| Gpr34 | 23890 | G Protein-Coupled Receptor 34 | Mm02620221_s1 | Lypsr1 |
| Ccl21b | 100042493 | Chemokine (C-C Motif) Ligand 21B | Custom see below | 6CKBAC1, 6Ckine, AI159701, CKb9, SLC, Scya21, Scya21a, TCA4 |
| Tlr2 | 24088 | Toll-Like Receptor 2 | Mm00442346_m1 | Ly105 |
| Dlg4 | 13385 | Discs, Large Homolog 4 | Mm00492193_m1 | Dlgh4, PSD-95, PSD95, SAP90, SAP90A |

| **Assay** | **reverse sequence** | **forward sequence** | **Probe** |
| --- | --- | --- | --- |
| CCl21b | TTCCTCAGGGTTTGCACATAG | CGAGGCTATAGGAAGCAAGAAC | /56-FAM/ATCCCGGCA/ZEN/ATCCTGTTCTTACCC/3IABkFQ/ |
