# Supplementary material for: Sexually divergent induction of microglial-associated neuroinflammation with hippocampal aging
Source: J Neuroinflammation. 2017 Jul 21;14:141. doi: 10.1186/s12974-017-0920-8 (PMC5521082; doi:10.1186/s12974-017-0920-8)
Supplement: Supplementary file 1 — Comparison of all pairwise gene expression sets. Figure S2. qPCR controls. Table S1. Cell-specific gene lists from Zhang et al. Table S2. Cell-specific gene lists from Zeisel et al. Table S3. Microglial gene lists from Hickman et al. Table S4. Gene Expression Assays. Table S5. Primary and secondary antibodies. Table S6. Transcripts differentially expressed with age. Table S7. Pathway, regulator and function changes with aging. Table S8. Transcripts differentially expressed between sexes. Table S9. Pathway, regulator and function differences between sexes. Table S10. Sex difference pathways, processes, and regulators. (ZIP 1133 kb) [file 12974_2017_920_MOESM1_ESM.zip › Additional files/Supplemental Table 6 -Antibodies.docx]

| **Specificity** | **Immunogen** | **Conc.** | **Host** | **Clonal** | **Supplier** | **Catalog** |
| --- | --- | --- | --- | --- | --- | --- |
| C1q – A | Synthetic peptide derived from within AA 50-150 of mouse C1q. | 1 ug/mL | rabbit | polyclonal | Abcam | Ab64632 |
| C1q – C | Peptide within internal region of mouse C1q-C | 1:500 | goat | polyclonal | Santa Cruz | SC-27669 |

C1q Immunoblotting Antibody list

| **Secondary Antibody** | **Host** | **Clonal** | **Supplier** | **Catalog** |
| --- | --- | --- | --- | --- |
| Rabbit Trueblot®: Anti-Rabbit IgG HRP | mouse | monoclonal | Rockland | 18-8816-31 |
| Goat Trueblot®: Anti-Goat IgG HRP | mouse | monoclonal | Rockland | 18-8814-33 |

C1 q Immunohistrochemistry Antibody List

| **Primary Antibody** | **Supplier** | **cat#** | **host** | **dilution** |
| --- | --- | --- | --- | --- |
| C1qa | Abcam (Generous gift from Dr. Ben Barres) | Ab4223 | Rabbit | 1ug/ml |
| Iba1 | Wako | 01919741 | Rabbit | 1:100 |
| C1q | Abcam | Ab71940 | Mouse | 1:100 |
| **Secondary Antibody** |  |  |  |  |
| Alexa fluor 488 donkey anti-rabbit IgG (H+L) | Invitrogen | A21206 |  | 1:500 |
| Alexa 488 donkey anti-rabbit (F(ab’)_2_; | Jackson ImmunoResearch | 711-546-152 |  | 1:200 |
| Alexa 647 donkey anti-mouse (F(ab’)_2_ | Jackson ImmunoResearch | 715-606-151 |  | 1:200 |
